# Potentially Useful Dwarfing or Semi-dwarfing Genes in Rice Breeding in Addition to the *sd1* Gene

**DOI:** 10.1186/s12284-022-00615-y

**Published:** 2022-12-21

**Authors:** Xiaoyan Cheng, Yongping Huang, Yong Tan, Lin Tan, Jianhua Yin, Guoxing Zou

**Affiliations:** 1 National Engineering Research Center of Rice (Nanchang), Rice Research Institute, Jiangxi Academy of Agricultural Sciences, Nanchang, 330200 Jiangxi People’s Republic of China; 2 Jiangxi Tiandao Liangan Seed Industry Co., Ltd., 568 South Huancheng Rd., Yuanzhou Dist., Yi Chun, 336000 Jiangxi People’s Republic of China

**Keywords:** Rice breeding, Dwarfing genes, Semi-dwarfing genes

## Abstract

The “Green revolution” gene *sd1* has been used widely in the breeding of modern rice varieties for over half a century. The application of this gene has increased rice yields and thereby supported a significant proportion of the global population. The use of a single gene, however, has raised concerns in the scientific community regarding its durability, especially given the bottleneck in genetic background and the need for large input of fertilizer. New dwarfing or semi-dwarfing genes are needed to alleviate our dependence on the sole “Green revolution” gene. In the past few years, several new dwarfing and semi-dwarfing genes as well as their mutants have been reported. Here, we provide an extensive review of the recent discoveries concerning newly identified genes that are potentially useful in rice breeding, including methods employed to create and effectively screen new rice mutants, the phenotypic characteristics of the new dwarfing and semi-dwarfing mutants, potential values of the new dwarfing and semi-dwarfing genes in rice breeding, and potential molecular mechanisms associated with the newly identified genes.

**Supplementary Information:**

The online version contains supplementary material available at 10.1186/s12284-022-00615-y.

## Background

Dwarfism is one of the most valuable traits in crop breeding, as it increases lodging resistance, improves crop yields, facilitates mechanical harvesting, and reduces microbe infection in fields (Evans 1993; Tar'an et al. 2003; Li et al. 2011; Xin et al. 2014). During the past half-century, the application of the dwarfing gene *Rht* in wheat and the semidwarf-1 (*sd1*) gene in rice triggered the so-called ‘Green Revolution’ in agriculture, resulting in dramatically elevated crop yields. Because of the Green Revolution, humans have largely avoided worldwide starvation during the great population expansion from four to approximately eight billion (Evans 1998; Hedden 2003).

The rice *sd1* gene, encoding a gibberellin 20-oxidase (OsGA20ox-2) (Ashikari et al. 2002; Sasaki et al. 2002; Spielmeyer et al. 2002), was originally derived from the Taiwanese variety Dee-Geo-Woo-Gen (DGWG) (Chandler 1968; Ferrero-Serrano et al. 2019). DGWG was first crossed with the Indonesian variety Peta, resulting in the semi-dwarf cultivar IR8. IR8 has produced dramatically higher yields per unit area and has formed the basis for the development of new high-yield, semi-dwarf rice cultivars since the 1960s (Institue 1967; Tomita 2009; Monna et al. 2002). At present, *sd1* remains one of the most important genes widely deployed in the world because of its rich polymorphism in nature (Spielmeyer et al. 2002; Asano et al. 2007; Peng et al. 2021).

Such extensive and worldwide use of a single gene will ineluctably result in limitations and potential problems. The situation has created a bottleneck for developing new rice varieties (Asano et al. 2009). To date, the rice yield potential (Evans 1993) has stagnated after the initial great leap in the ‘Green Revolution’ and the development of hybrid technology (Peng et al. 2008; Siddiq and Vemireddy 2021). The genetic uniformity also makes rice vulnerable to genetic erosion and sudden outbreaks of crop pests, diseases, or other abiotic threats (Siddiq and Vemireddy 2021; Asano et al. 2009). In addition, the use of *sd1* requires heavy utilization of nitrogen fertilizers to achieve high yields. Constant use of high levels of nitrogen fertilizers threatens the diversity and sustainability of ecosystems and agricultural systems. Widespread and extensive use of nitrogen fertilizers also results in a huge increase in production costs (Wang et al. 2021a; Li et al. 2021). Currently, the nitrogen input of rice with the *sd1* gene has been decreased by regulation of GROWTH-REGULATING FACTOR 4 (GRF4) in the laboratory (Li et al. 2018), but the practical application of GRF4 in the field is still being developed. GRF4 could simultaneously increase rice storage capacity and decrease seed shattering, both of which are highly desirable in modern rice breeding (Sun et al. 2016). Furthermore, the increasing rice-growing area with cultivars containing *sd1* evokes another problem that of genetic erosion (Frankel 1970; Khoury et al. 2022; Harlan 1975). Recently, gene editing based on the clustered regularly interspaced short palindromic repeats (CRISPR)/CRISPR-associated protein9 (Cas9) system has been used to edit *SD1* in rice cultivars. This new method can relieve genetic erosion in modern rice varieties, but more investigation is needed before the CRISPR/Cas9 system can be applied in rice molecular breeding (Chen et al. 2019; Hu et al. 2019; Biswas et al. 2020).

To overcome these limitations and potential problems, we need to enrich the dwarfing (semi-dwarfing) gene pool and identify additional dwarf and semi-dwarf genes that are suitable for practical use in rice breeding (Asano et al. 2009; Liu et al. 2018). A great deal of effort has been put into searching for new rice dwarf (semi-dwarf) mutants through various methods, but to date limited progress has been achieved (Peng et al. 2021). Here, we summarize recent advances on new dwarf (semi-dwarf) mutants and genes in the literature. We focus on the methods used to identify new mutants, their phenotypic characteristics including cytological characteristics, the potential value in rice breeding, and potential molecular mechanisms associated with the newly identified genes.

## Methods of Identifying New Dwarfing/Semi-dwarfing Mutants

Generation of artificial mutations is considered as one of the most effective methods to discover new genetic resources for practical breeding. Rice artificial mutants can be generated by chemical reagents or radiation. Mutants *Slr1-d1*, *Slr1-d2*, *Slr1-d3*, *Slr1-d4, D-h*, and *Tid1* resulted from treatment of rice with MNU (Asano et al. 2009, Hirano et al. 2010, KOH and HEU 1993, Sunohara et al. 2009). *Slr1-d5* was generated using ethyl methane sulfonic acid (EMS) (Zhang et al. 2016). Physical radiation has also been utilized to produce new rice mutants. KL908 resulted from γ-irradiation (Wei et al. 2006), and Y98149 and Sdd were obtained using low-energy nitrogen ions (Liu et al. 2008a, 2008b). The DMF-1 mutant was obtained by X-ray (Sunohara et al. 2006). Tissue culture is a conventional tool employed to induce mutations (Jiang and Ramachandran 2010). For example, 986083D originated from the anther culture of an autotetraploid indica/japonica hybrid (Qin et al. 2008), and SV14 was obtained through somaclonal variation of a thermo-sensitive male sterile line (Liu et al. 2018). Wild rice is a good source of natural mutants that are useful in breeding (Sakai and Itoh 2010; Zhao et al. 2018). Recently, gene editing using the CRISPR/Cas9 system has also been used for generating mutants and identifying useful genes. Application of this cutting-edge technology has created semi-dwarf mutants *slr1-d7, slr1-d8, slr1-d9, slr1-d10, slr1-d11*, and *slr1-d12* (Jung et al. 2020). Although natural mutation occurs at low frequency in higher plants, large and advanced backcross populations have been employed to produce new rice mutants (Jiang and Ramachandran 2010), including the spontaneous mutants *Slr1-d5*, *Shennong9816d*, *sdt,* and LB4D (Fan et al. 2016; Zhao et al. 2015; Wu et al. 2018; Liang et al. 2011). Researchers have also used space mutagenesis to create new crop germplasm. For example, CHA-2 was screened from the progeny of an *indica* TXZ13 after space-induced mutations (Guo et al. 2011).

## Phenotypic Dwarfism of the Newly Identified Mutants

**Fig. 1:**
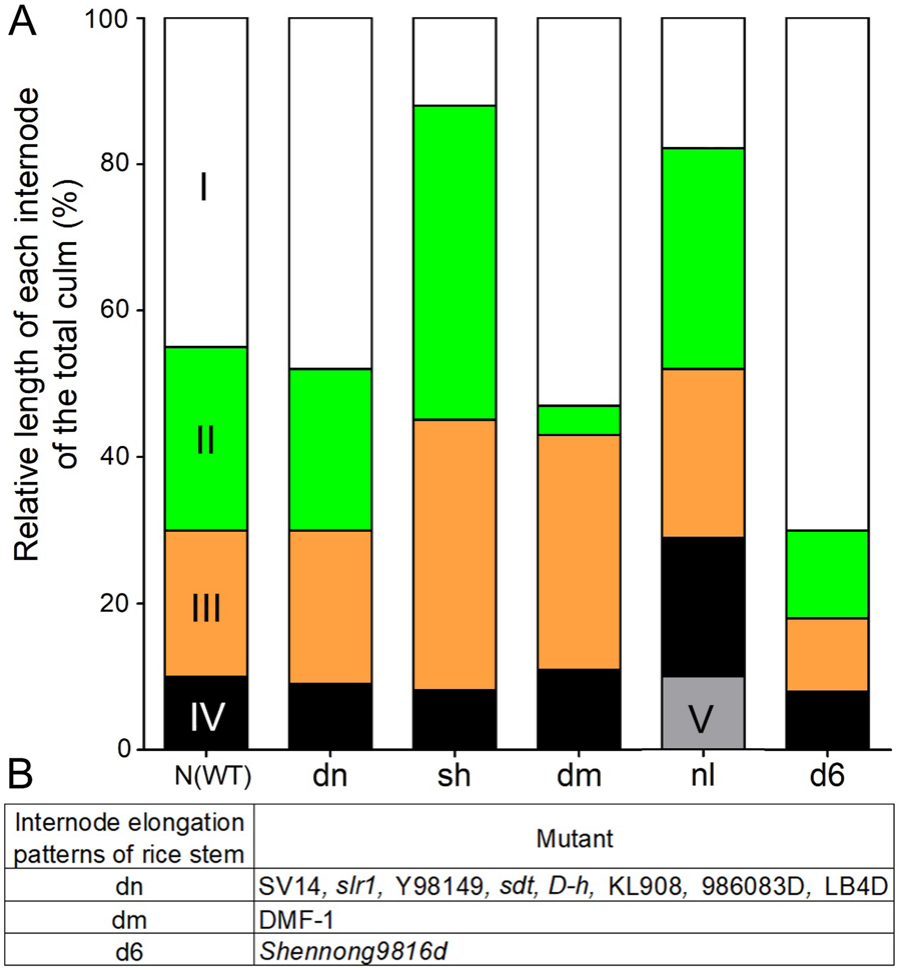
Internode elongation patterns of the newly identified mutants. **A** Schematic diagram of internode elongation patterns of rice stem (redrawn from Takeda 1977). I: the first internode, shown in white block; II: the second internode, shown in green block; III: the third internode, shown in orange block; IV: the fourth internode, shown in black block; V: the fifth internode, shown in grey block. **B** The newly identified mutants with different internode elongation patterns

The rice culm usually includes 4–6 elongated internodes in a mature plant. Dwarf and semi-dwarf plants show various reductions in the internodes. Internodes on rice plants are counted from the top to the base at the mature stage. According to internode elongation patterns, dwarfing rice plants are classified into six groups: N-type, dn-type, sh-type, dm-type, nl-type, and d6-type (Takeda 1974, 1977); these are schematically representated in Fig. 1A. N-type plants elongate normally between all internodes like wild-type plants. All internodes are uniformly reduced in dn-type mutants. Plants of the other four mutant types exhibit reduction in elongation of a specific internode or combinations of nodes. Specifically, sh-type mutants exhibit reduction in the first internode; dm-type mutants exhibit reduction in the second internode. For nl-type mutants, the fourth internode is relatively longer, while the first internode is much shortened. For the d6-type mutants, all of the internodes are shortened except for the first internode (Takeda 1977).

As shown in Fig. 1B, newly identified dwarfing and semi-dwarfing mutants, including SV14, *slr1*, Y98149, *sdt*, *D-h*, KL908, 986083D, and LB4D, are dn-type, with all internodes shortened (Liu et al. 2018, 2008a; Asano et al. 2009; Zhang et al. 2016; Jung et al. 2020; Wu et al. 2018; Zhao et al. 2015; Piao et al. 2014; Wei et al. 2006; Qin et al. 2008). DMF-1 is the only identified mutant that is considered a dm-type, with a shortened second internode (Miura et al. 2009; Wu 1997) while *Shennong9816d* is considered a d6-type (Fan et al. 2016).

Cytological studies have revealed that internode length reduction in plants with a dwarfing or semi-dwarfing phenotype is due to either shortening of stem cell lengths, reduced numbers of cells, or a combination of both shortened cell lengths and reduced cell numbers (Liu et al. 2018; Jung et al. 2020; Sunohara et al. 2009; Piao et al. 2014; Wu et al. 2018; Liang et al. 2011; Zhou et al. 2013; Zhao et al. 2018). Interestingly, the stem cells of the mutant *sdt* are actually longer than those of the wild type, and the shortened internodes in this mutant are the result of exclusively reduced cell numbers. Based on these results, the functions of dwarfing-related genes may involve negative regulation of cell growth in the stem (Zhao et al. 2015).

## Potential Use of the Newly Identified Dwarfing/Semi-dwarfing Genes

**Fig. 2:**
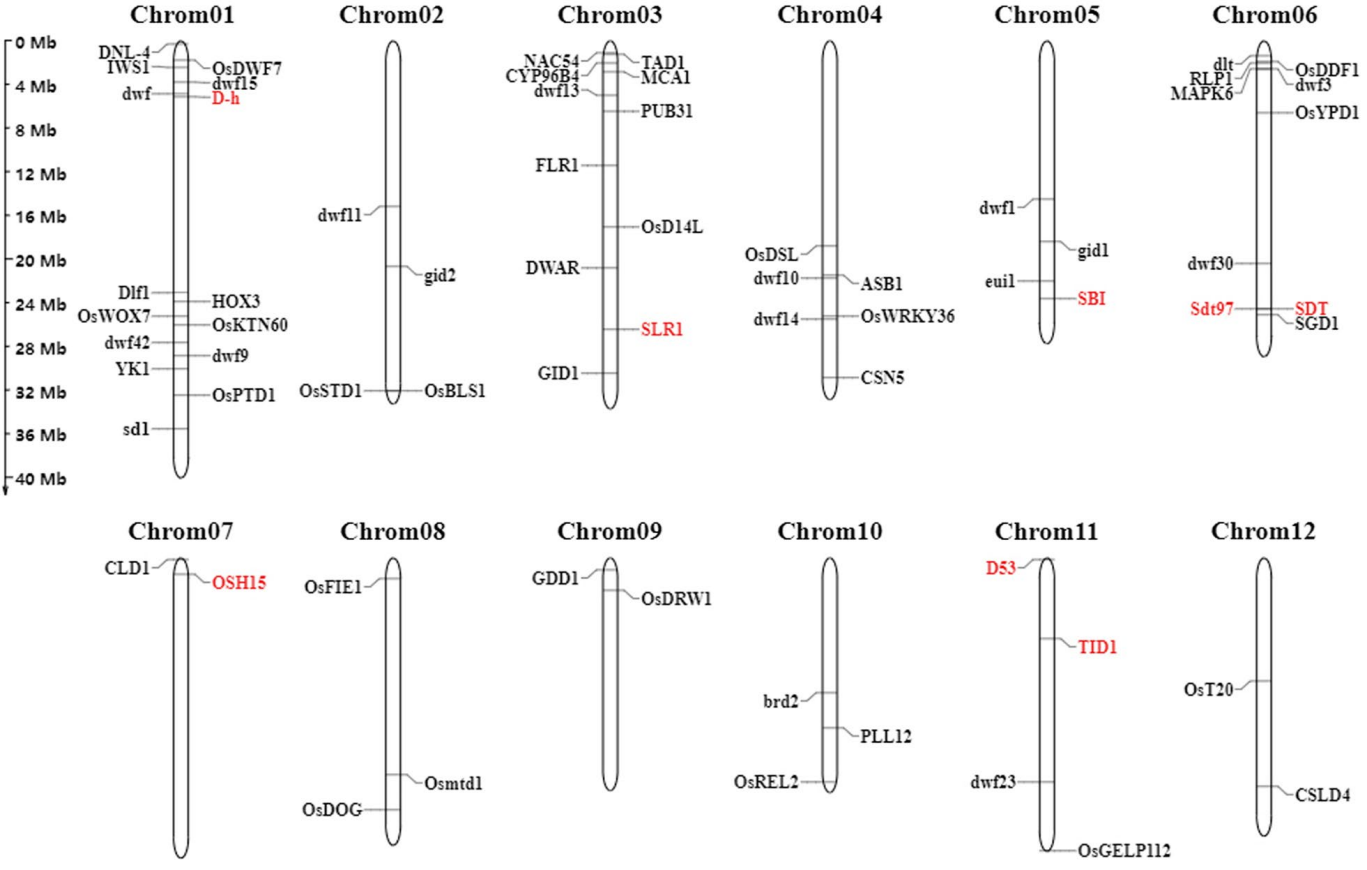
Sixty-six dwarf and semi-dwarf genes obtained from the Rice Annotation Project Database (https://rapdb.dna.affrc.go.jp/). The genes in red are discussed in the following text. The chromosomal locations of these genes are determined using the program Map Gene 2 Chromosome V2 (MG2C) (Chao et al. 2021)

To date, more than 60 dwarf and semi-dwarf mutants have been identified from *indica* and *japonica* rice (Futsuhara and Kikuchi 1997; Gaur et al. 2020; Peng et al. 2021). We used both ‘dwarf’ and ‘semi-dwarf’ as the key words to search the Rice Annotation Project Database (https://rapdb.dna.affrc.go.jp/), and we found 66 dwarf and semi-dwarf genes, as shown in Fig. 2 (details in Additional file 1: Table S1). Although these genes are dispersed throughout the rice genome, chromosomes 1, 3, and 6 contain the most genes, with 15, 11, and 10 genes on these chromosomes, respectively. In addition, *Ssi1*, *Dd7*, *Dx* and *LB4D* were not shown in Fig. 2, because the gene loci have not been verified. Most of the genes identified from the mutants may not be suitable for breeding due to their unfavorable agronomic phenotypes, such as severe dwarf, low fertility, and short grain (Nagai et al. 2018; Wu et al. 2018).

Rice mutant *D-h* with a semi-dwarf phenotype was obtained from the Korean j*aponica* cultivar Hwachung after being treated with MNU (KOH and HEU, 1993). The mutant phenotype is linked with a 63-bp deletion in the *d-h* locus flanked by the two STS markers S3197-1 and D-h-3 on the short arm of chromosome 1 (Piao et al. 2014). The *D-h* gene was predicted to take part in an unknown GA signaling pathway that made the mutant insensitive to GA. The *D-h* mutant also exhibits both short compact panicles and small round grains, making it unsuitable for hybrid breeding (Piao et al. 2014). However, the dominant dwarf gene can facilitate rice breeding by avoiding using a recessive gene for both male and female parents (Piao et al. 2014; Asano et al. 2009).

X-ray irradiation was also used to induce mutations in the *japonica* cultivar Fujiminori to identify dwarf mutants. A new dominant semi-dwarf mutant named DMF-1 was created from irradiation (Yamaguchi 1976). A dominant gene named *Ssi1* (*Short second internode 1*) was identified by this method. *Ssi1* was mapped to a 7.3-cM region flanked by molecular markers M45 and M26 on the long arm of chromosome 1 (Wu 1997; Miura et al. 2009; Sunohara et al. 2006). A 1.3-Mbp genomic inversion around the *Ssi1* locus reduced the second internode elongation in DMF-1 (Sunohara et al. 2006; Miura et al. 2009). The *Ssi1* appears to be involved in brassinosteroid (BR) metabolism to reduce the rice plant height (Miura et al. 2009).

The newly identified *SLR1* gene with the full name *DELLA protein SLENDER RICE1* also functions in gibberellic acid (GA) metabolism. SLR1 acts as a repressor of GA signaling, as SLR1 degradation could trigger most of the GA-associated responses in rice (Hirano et al. 2010). Six alleles of natural *Slr1-d* mutants have been identified, namely, *Slr1-d1*, *Slr1-d2*, *Slr1-d3*, *Slr1-d4*, *Slr1-d5*, and *Slr1-d6*. These mutants resulted from gain-of-function mutations via amino acid substitutions. *Slr1-d1*, *Slr1-d3*, *Slr1-d5*, and *Slr1-d6* have single amino acid substitutions in the TVHNP domain, *Slr1-d2* in the DELLA domain, and *Slr1-d4* in the GRAS domain (Wu et al. 2018; Hirano et al. 2010; Asano et al. 2009; Zhang et al. 2016). *Slr1-d1* and *Slr1-d3* are considered unsuitable for breeding due to their association with severe dwarf phenotype and low fertility, respectively (Asano et al. 2009). Rice lines with *Slr1-d2* have shown suitable culm length, indicating that this gene may be useful for breeding (Asano et al. 2009). When *Slr1-d5* was introduced into the *indica* variety 93–11, homozygous plants showed 30% reduction in plant height. Additionally, plants containing *Slr1-d5* displayed large increases in the number of tillers that may contribute to higher yields (Zhang et al. 2016). When *Slr1-d6* was introduced into the genetic background of 93–11, it reduced plant height by 37% but still maintained an acceptable seed setting rate for sterile line production (Wu et al. 2018). *Slr1-d6* heterozygous plants resulted in a 25% increase in yield compared with *sd1* semi-dwarf 93–11 (Wu et al. 2018). Thus, *Slr1-d2*, *Slr1-d5*, and *Slr1-d6* hold great promise in rice breeding. Numerous studies on the application of these genes are currently underway (Asano et al. 2009; Zhang et al. 2016; Wu et al. 2018). More recently, allelic variants named as *slr1-d7*, *slr1-d8*, *slr1-d9*, *slr1-d10*, *slr1-d11*, and *slr1-d12* were created by gene editing using the CRISPR/Cas9 system. All six mutants showed nucleotide mutations in the TVHYNP domain. Specifically, *slr1-d7*, *slr1-d8*, *slr1-d9*, and *slr1-d10* were identified with bp deletions, while *slr1-d11* and *slr1-d12* had one bp insertion (Jung et al. 2020). After initial testing, rice lines containing *slr1-d7* and *slr1-d8* were considered potentially valuable in rice breeding as semi-dwarf germ resources (Jung et al. 2020).

One of the newly identified dwarf genes is the *Shortened Basal Internodes* (*SBI*) gene on chromosome 5. *SBI* is a semi-dominant gene encoding a gibberellin 2-oxidase that specifically controls the elongation of culm basal internodes by reducing the amounts of active gibberellins (GAs) in the tissue (Liu et al. 2018). *SBI* was identified from the cultivar SV14, a progeny of the *indica* male sterile line Zhu1S with somaclonal variation (Liu et al. 2002, 2018). Initial evaluation of over 94 varieties carrying *SBI* revealed strong lodging resistance in comparison with their parent lines. Among these evaluated *SBI* cultivars, Lingliangyou 22 and Lingliangyou 211 were the best based on their lodging resistance, yield, and grain quality. These two cultivars have been planted in over 366,000 ha in China (Liu et al. 2018).

An allelic variant of *SEMIDWARF AND HIGH-TILLERING* (*SDT*) was mapped to the long arm of chromosome 6 after a genetic analysis on one chromosome segment substitution line (CSSL) derived from a natural rice *semi-dwarf and high-tillering* (*sdt*) mutant (Zhao et al. 2015). The *sdt* allele is able to regulate the polyadenylation of the *Osmi156h* microRNA precursor. Thus, the *sdt* mutant exhibits both increased lodging resistance and tiller number, factors that may contribute to the harvest index and grain productivity (Zhao et al. 2015). When both *sdt* and *sd1* were pyramided, the hybrid rice yield increased by about 20% (Zhao et al. 2015).

Rice mutant Y98149 has a semi-dwarf phenotype and was identified from the progeny of the *japonica* rice variety Y98148 (Wu et al. 2002; Liu et al. 2008b). The mutant phenotype is controlled by a new dominant gene designated as *Sdd(t)*. *Sdd(t)* has been mapped to the short arm of chromosome 6 between two STS (sequence-tagged site) markers S9 and S13 (Liu et al. 2008a), and *Sdd(t)* was later found to be identical to *Sdt97* (Tong et al. 2001; Tong et al. 2007). With a residual heterozygous line (RHL) population, this novel gene was mapped to the long arm of chromosome 6 flanked between STS marker N6 and SNP marker N16 (Tong et al. 2016). The *Sdt97* gene can alter the transcription level of methyladenine glycosylase to regulate GA synthesis (Tong et al. 2007, 2016). Researchers have successfully combined *Sdt97* and the photoperiod-sensitive male sterile gene *pms3* to develop a series of semi-dwarf photoperiod-sensitive genic male-sterile (PSGMS) rice varieties, thereby providing a powerful genetic tool in two-line hybrid rice breeding systems (Tong et al. 2007, 2016).

Fan et al. (2016) identified a spontaneous mutant, *Shennong9816d*, from the rice *japonica* variety Shennong9816. The mutant had only 60.42% of the wild-type plant height. Genetic analysis demonstrated that a recessive gene *osh15(t)* near the short arm of chromosome 7 was responsible for the dwarfing phenotype. The *osh15(t)* gene is an allele of *OSH15* that affects the architecture of internodes and leads to rice dwarfism (Sato et al. 1999). The *Shennong9816d* mutant showed decreased total number of grains per panicle, but increased tiller number compared with the wild type. Interestingly, the mutant *Shennong9816d* contains higher amounts of chlorophyll than the wild type.

Zhao et al. (2018) discovered a dominant dwarfing gene, *Dd7*, from crosses between the *japonica* cultivar Dianjingyou 1 (DJY1) and the African wild rice *Oryza bathii*. *Dd7* is considered a potential valuable source for rice breeding based on its various favorable agronomic traits, such as number of grains per panicle, heading date, and 1000-grain weight. *Dd7* was mapped to an 82-kb region on chromosome 7 with 14 open reading frames (ORFs). *Dd7* is thought to be involved in abscisic acid (ABA) metabolism other than GA or BR biosynthesis/signaling pathways to regulate the rice dwarf phenotype.

Qin et al. identified a dominant dwarf mutant 986083D (*japonica*) from the progeny of the anther culture of an autotetraploid *indica/japonica* hybrid. A dominant dwarf gene named *Dx* was mapped onto the short arm of chromosome 8 flanked by the SSR markers RM6356 and RM6208. *Dx* altered the rice dwarf phenotype neither through GA metabolism or signaling nor by SL signaling (Qin et al. 2008). The molecular mechanism remains unknown. Plant heights of F_1_ crosses with 986083D displayed 40% to 53.5% reduction in a subtropical environment and with 37.5% to 48.2% reduction in a temperate environment (Qin et al. 2008). *Dx* may be a valuable material in rice breeding in the future (Qin et al. 2008).

Iwata et al. identified a mutant named KL908 in 1977 from irrated *japonica* variety NL8. Later, a dominant dwarfing gene, *D-53*, was identified from this mutant (Kinoshita 1984). *D-53* was mapped onto the short arm of chromosome 11 and encodes a protein that belongs to the nucleoside triphosphate hydrolase superfamily with a double Clp-N motif-containing a P-loop (Wei et al. 2006; Jiang et al. 2013; Zhou et al. 2013). D-53 acts as a repressor in strigolactone (SL) signaling, and thus controls the dwarf phenotype in this mutant (Zhou et al. 2013; Jiang et al. 2013).

Sunohara and Kitano (2003) identified a mutant named *Twisted dwarf 1* (*Tid1*) from the *japonica* cultivar Taichung 65 (T65) after being mutagenized with MNU. *TID1* was mapped onto the short arm of chromosome 11 and encodes an α-tubulin protein. Mutated *TID1* resulted in defective microtubules, leading to severe dwarfism and reduced right helical growth (Sunohara et al. 2009). Even though homozygous *Tid1* mutants are lethal or sterile (Sunohara and Kitano 2003; Qin et al. 2008), the mutant may still be useful in practical rice breeding due to its semi-dominant characteristics.

Liang et al. (2011) idenfied a spontaneous dwarf mutant named LB4D from the progeny of wild rice backcrosses while constructing wild rice introgression lines. The LB4D mutant contains a single semi-dominant dwarf gene (*LB4D*) with a strong dwarfing effect in plants under different genetic backgrounds. *LB4D* reduced plant height in F_1_ plants from 27.9 to 38.1%. *LB4D* was mapped to a 46-kb region between markers Indel 4 and Indel G on the short arm of chromosome 11. The gene *LB4D* was supposed to be independent of GA biosynthesis and signaling pathways. Further studies on the molecular mechanism underlying rice height are needed. Based on its overall favorable characteristics, *LB4D* may be valuable for ‘two-line’ hybrid rice breeding.

At present, two genes, *Sdt97/Sdd(t)* and *SBI* from the mutants Y98148 and SV1, respectively, have begun to exhibit their breeding value. Mutant Y98149 reduced plant height by about 28% with a slightly shorter panicle length compared with the parental cultivar Y98148 (Liu et al. 2008a). *Sdt97/Sdd(t)* and *pms3* have been combined to develop a series of semi-dwarf PSGMS rice varieties, and they are now available for a two-line hybrid rice breeding program (Tong et al. 2007, 2016). Heterozygous SV1 plants showed a slightly reduced seed setting rate and number of filled grains per panicle but greatly increased effective numbers of panicles per plant and 1000-grain weight, with overall improved yield (Liu et al. 2002). The *SBI* allele was introduced into various rice cultivars to breed more than 94 authorized new varieties that were confirmed as having enhanced lodging resistance as well as higher grain yield. Notably, two new varieties were considered as the elite rice varieties that are now planted in over 360,000 ha in China (Liu et al. 2018).

## Genetic and Molecular Mechanisms of the Newly Identified Genes

Dominant and semi-dominant dwarfing genes are considered more valuable in hybrid crop breeding compared with recessive genes. For recessive genes such as *sd1,* lodging resistance only occurs when both parents carry the same recessive gene (Peng et al. 2021). For dominant or semi-dominant dwarfing genes, only one parent needs to have the gene for lodging resistance; this makes hybrid breeding much easier (Qin et al. 2008; Chen et al. 2018). The availability of newly identified dominant or semi-dominant dwarfing genes should reduce the problems associated with the extensively used recessive *sd1* gene (Asano et al. 2009). Accordingly, researchers have focused on identifying dominant and semi-dominant dwarfing mutants during the past two decades. As a result, each the newly identified genes except one were dominant or semi-dominant (Liang et al. 2011; Sunohara et al. 2009, 2006; Qin et al. 2008; Wei et al. 2006; Piao et al. 2014; Zhao et al. 2015, 2018; Tong et al. 2016; Liu et al. 2008a, 2018; Asano et al. 2009).

The dominant or semi-dominant effects of these genes were unrelated with their capability to reduce plant height when they were introduced into various rice varieties. Zhu et al. reported that the plant height of the F_1_ generations from KL908 and other high rice varieties was close to or slightly greater than the midparent value, confirming that the dominant effect of *D53* in the KL908 is quite weak (Zhu et al. 1995). The semi-dwarfing ability of *Slr1-d6* has been demonstrated to be stronger than *sd1* when they are in the same genetic background of *indica* 9311, while the culm length of *Slr1-d6* heterozygote plants was similar to that of the 9311 with *sd1* (Wu et al. 2018). Similarly, dominant *Dd7* showed relatively weak ability to reduce the plant height in F_1_ hybrid plants (Zhao et al. 2018). The dominant reduction of plant height from *Slr1-d5* homozygous plants was 30%, a value that was recognized as a mild level when *indica* 9311 was backcrossed with *Slr1-d5* (Zhang et al. 2016). Another semi-dominant gene, *LB4D,* was able to reduce the plant height from 27.9 to 38.1% after being introduced into different rice varieties, showing a strong dwarfing effect (Liang et al. 2011). *Dx* was considered the strongest dominant dwarfism effect gene to date, since the plant height reduction of the F_1_ hybrids varied from 37.5 to 53.5% in different environments after crosses with various rice cultivars (Qin et al. 2008). As for the recessive dwarf gene *osh15(t)* in mutant *Shennong9816d*, the gene reduced plant height by 39.58% of compared with the wild type (Fan et al. 2016).

The newly identified dwarfing and semi-dwarfing genes usually affect other phenotypes in addition to plant height. Their pleiotropic characteristics make their usefulness in rice breeding more dependent on the balance between the positive contribution of dwarfism and the potential negative impact on other important agronomic traits. The genes with the most dramatic reduction in plant heights include *Slr1-d1*, *Slr1-d2*, *Slr1-d3*, *Slr1-d4*, *Slr1-d5*, *Slr1-d8*, KL908, *Tid1-1*, and *Shennong9816d*. These genes can reduce plant height by more than 50% compared to the wild types (Wu et al. 2018; Asano et al. 2009; Zhang et al. 2016; Hirano et al. 2010; Wei et al. 2006; Fan et al. 2016). Most of these genes, however, may not be useful in breeding unless their unfavorable agronomic characteristics can be overcome. For example, the culm lengths of the mutants *Slr1-d1* and *Slr1-d2* are too short for developing sterile lines for rice breeding, even though no other unfavorable phenotypes are associated with these genes (Asano et al. 2009). Fertility is too low for rice lines carrying the *Slr1-d3* gene to be useful in breeding (Asano et al. 2009). Mutants carrying *Slr1-d8* have shorter panicles with decreased grains per plant (Jung et al. 2020). Mutant KL908 had significantly decreased tillers, smaller panicles, and thinner stems (Wei et al. 2006). Homozygous plants with *Tid1-1* are frequently lethal or sterile, and they are also too short (only 2 cm plant height) (Sunohara and Kitano 2003). *Tid1-1* heterozygous plants exhibit much milder dwarfism but have significantly shorter roots (Sunohara et al. 2009). *Shennong9816d* plants have increased chlorophyll content and greater numbers of tillers. However, the lower 1000-grain weight together with reduced fertility and smaller grains mean that *Shennong9816d* is not useful in breeding without further improvement (Fan et al. 2016). Heights of the plants with the dominant gene *D53* vary greatly once this gene is introduced into other rice varieties (Zhu et al. 1995; Qin et al. 2008). Therefore, by far the most promising gene for rice breeding among the genes with dramatic reduction in plant heights is the gene in the mutant *Slr1-d5*. *Slr1-d5* homozygous lines resulted in 30% reduction in plant height when the *indica* variety 93–11 was backcrossed with mutant *Slr1-d5* (Zhang et al. 2016).

The genes with milder dwarfism include *Slr1-d6*, *Slr1-d7*, *Ssi1*, and *Dd7*. These genes reduce plant height by around 10–20 cm, resulting in a favorable condition for plants to catch pollen during pollen shedding from female panicles for hybrid rice fertilization (Wu et al. 2018; Jung et al. 2020; Sunohara et al. 2006; Zhao et al. 2018). *Slr1-d6* plants show no significant yield penalty. In fact, *Slr1-d6* plants have significantly increased effective tillers per plant. However, this positive effect is partially offset by decreases in spikelets per panicle and 1000-grain weight during seed setting. The usefulness of *Slr1-d6* in rice breeding comes from yield increase in heterogenous plants. When *Slr1-d6* was introduced into rice variety 9311, heterozygous plants exhibited a 25% yield increase (Wu et al. 2018). Unlike *Slr1-d6* that has shown potential usefulness in rice breeding, *Slr1-d7* has several undesirable agronomic traits and needs significant improvement to have any practical value. Even though *Slr1-d7* plants have thickened internodes (Jung et al. 2020) that makes it possible to support larger panicles with more grains, these plants have shorter panicles with reduced grains and withered leaves. A similar situation was found with plants carrying *Slr1-d8* (Jung et al. 2020). Defects with *Ssi1* plants include a high ratio of hull-cracked kernels that leads to low grain quality and reduced yield (Sunohara et al. 2006). The semi-dominant gene *Ssi1* underlying the dm-type internode elongation pattern can be stably transferred to different genetic backgrounds, but only if it is in a homozygous status, a factor that further limits its usefulness in rice breeding. *Dd7* from the wild rice *Oryza barthii* introduced into NIL produced no visible defects in agronomic traits except for the smaller number of grains per panicle that was compensated by the higher panicle number. Plants with *Dd7* have overall improved grain yield (Zhao et al. 2018).

The *LB4D* and *Dx* genes produce effects that fall between genes causing dramatic plant height reduction and genes causing mild plant height reduction. Plant heights with *LB4D* or *Dx* also vary greatly under different genetic or environmental conditions. Heterozygous *LB4D* plants had 27.9–38.1% plant height reduction. Heterozygous *Dx* plants in various genetic backgrounds had 37.5–48.2% plant height reduction in a temperate environment and 40–53.5% reduction in a subtropical environment. *LB4D* decreased tiller number and average grain weight (Liang et al. 2011). *Dx* is a strongly dominant gene. Both homozygous and heterozygous *Dx* plants share similar phenotypes, including decreased fertility and increased tillers (Qin et al. 2008).

Most cloned dwarf (semi-dwarf) genes play important roles in the biosynthesis and/or response to three plant hormones, gibberellins (GAs), brassinosteriods (BRs), and strigolactones (SLs) (Zhang et al. 2016; Fan et al. 2016). GAs are a large family of natural tetracyclic diterpenoid carboxylic acids that function as regulators of plant growth and development. To date, 136 GAs have been isolated from higher plants, fungi, and bacteria (Hedden 2017). Rice mutants defective in GA metabolism or signaling pathways normally exhibit proportional dn-type shortened internodes (Zhang et al. 2016; Fleet and Sun 2005; Sakamoto et al. 2004; Wu et al. 2018). The gene named *Shortened Basal Internodes* (*SBI*) from mutant SV14 encodes a gibberellin 2-oxidase designated as *GA2ox4*. This enzyme converts active GA_1_, GA_9_, and GA_20_ into inactive GA_8_, GA_51_, and GA_29_ (Liu et al. 2018). As a result, the abundance of GA_1_ decreases and GA_29_ increases in the tissues of internodes of the mutant SV14, resulting in the dn-type dwarf (Liu et al. 2018). The GA signaling pathway includes GA receptor GA-INSENSITIVE DWARF1 (GID1) (Ueguchi-Tanaka et al. 2005), the F-box protein GA-INSENSITIVE DWARF2 (GID2) (Sasaki et al. 2003; Gomi et al. 2004), and DELLA protein SLENDER RICE1 (SLR1) (Ikeda et al. 2001). The DELLA protein SLR1 acts as a transcription regulator, suppressing the expression of intracellular GA-response genes in the absence of GA (Ogawa et al. 2000; Ikeda et al. 2001). In the presence of GAs, SLR1 binds with GID1 and stimulates the formation of a GA-GID1-DELLA complex that triggers the degradation of SLR1 via the SCF^GID2^-proteasome pathway, leading to transcriptional activation of GA-responsive genes (Sun and Gubler 2004; Eckardt 2007; Ikeda et al. 2001). Natural single amino acid substitutions or artificial deletion/insertion mutants in the functional motifs of DELLA protein led to similar dn-type dwarf *slr1* mutants. The *slr1* mutants were defective in GA signaling, resulting in dwarfism (Wu et al. 2018; Zhang et al. 2016; Jung et al. 2020; Asano et al. 2009). The *d-h* gene in the mutant *D-h* was predicted to participate in an unknown GA signaling pathway, since the expression levels of the genes in the GA biosynthetic and signaling pathways were conflicting (Piao et al. 2014). The exact mechanism of *d-h* mediated dwarfism remains unclear (Piao et al. 2014).

To investigate the relationship between GA and dwarfism, researchers also classified rice plants into four groups: N, T, D, and E based on two physiological processes: elongation of shoots and active α-amylase activity in the endosperm. Group N (normal type) usually includes normal cultivars and semidwarf mutants. The group N plants exhibited a slightly increased elongation of shoots and a much greater increase in α-amylase activity after exogenous GA_3_ treatment. Members of group T (Tan-ginbozu type) are often GA-deficient mutants that are highly responsive to exogenous GA_3_ in terms of elongation of shoots and production of α-amylase, even with minimum levels of endogenous GA treatment. Group D (Daikoku type) plants are GA-insensitive and are not responsive to exogenous GA_3_. Group E plants show responses to exogenous GA_3_ similar to group N plants (Mitsunaga et al. 1994). For example, mutant 986083D, a group D mutant, is moderately sensitive to exogenous GA_3_ based on the elongation of shoots as well as induction of α-amylase activity in the endosperm (Qin et al. 2008). Mutations in the functional motifs of DELLA protein SLR1 resulted in a set of *slr1* mutants defective in GA signaling. Thus, these *slr1* mutants are group D members (Wu et al. 2018; Zhang et al. 2016; Jung et al. 2020; Asano et al. 2009). The mutants Y98148 and LB4D exhibited similar responses to exogenous GA_3_ as their wild types, but the induction of α-amylase from half-seeds without embryos and the shoot length did not differ significantly from the corresponding wild types. Therefore, these mutants were considered as group E (Liang et al. 2011; Liu et al. 2008a). Mutant *Shennong9816d* is considered a group T member because it is sensitive to GA_3_ but deficient in GA synthesis (Fan et al. 2016). The reduced plant heights of *Shennong9816d* mutants are due to the inhibited elongation of the bottom internodes (d6-type dwarfism) (Fan et al. 2016).

BRs are a specific class of endogenous polyhydroxysteroids essential for plant growth and development (Li 2013). Defects in the BR biosynthesis or signaling pathway lead to mutants with pleiotropic aberrant phenotypes such as dwarfism, erect leaves, and small grains (Piao et al. 2014; Fan et al. 2016; Hong et al. 2002, 2005). Rice dm-type mutants are usually BR-deficient and -insensitive mutants (Hong et al. 2005; Hu et al. 2013; Zhang et al. 2014; Yamamuro et al. 2000). Key components in rice BR biosynthesis include OsDWARF, Dim/dwf1, D2/CYP90D2, SG4, CYP90B2/OsDWARF4, and D11/CYP724B1, as revealed from characterizing the mutants *brassinosteroid-dependent 1* (*brd1*), *BR-deficient dwarf2* (*brd2*), *ebisu dwarf* (*dwarf2* or *d2*), *small grain 4* (*sg4*), and *osdwarf4-1*, *dwarf11*, respectively (Hong et al. 2002, 2005, 2003; Shi et al. 2015; Sakamoto et al. 2006; Tanabe et al. 2005). The rice BR signaling pathway is overall similar to that of *Arabidopsis*, with conserved proteins such as BRI1, BAK1, GSK1, BZR1, and BIN2 (Zhang et al. 2014; Fàbregas and Caño-Delgado 2014; Tong and Chu 2012; Nakagawa et al. 2011). In rice plants, however, there is another G-protein signaling pathway involved in BR signaling via the U-box E3 ligase TAIHU DWARF1 (TUD1) that interacts with the rice Gα subunit D1 (Nakagawa et al. 2011; Hu et al. 2013; Oki et al. 2009a, 2009b). The D1-TUD1-mediated BR signaling pathway functions parallel to or overlapping with the typical BRI1 pathway in regulating the elongation of the second internode (Hu et al. 2013). The elongation of the second internode is also regulated by *Ssi1* (Miura et al. 2009).

SLs are a class of root-derived terpenoid lactones involved in regulating plant development and growth features such as shoot architecture, stem secondary growth, seed germination, root development, leaf senescence, and photomorphogenesis as well as responding to environmental stresses such as low-phosphate and nitrate deficiency (Mashiguchi et al. 2021; Seto et al. 2012; Brewer et al. 2013). Biosynthesis of active SLs involves many proteins and enzymes, including D10, HTD1/D17, D27, and HTD2 (Arite et al. 2007; Lin et al. 2009; Zou et al. 2006; Wang et al. 2020; Liu et al. 2009). D14 is an α/β-fold hydrolase that serves as a receptor for active SLs (Arite et al. 2009; Gao et al. 2009; Hamiaux et al. 2012; Jiang et al. 2013; Hu et al. 2017). D3 is an F-box leucine-rich repeat protein that plays a central role in SL signaling (Ishikawa et al. 2005; Hu et al. 2017; Jiang et al. 2013; Zhou et al. 2013). D53 belongs to the superfamily of double Clp-N motif-containing, P-loop nucleoside triphosphate hydrolases and functions as a repressor of SL signaling (Zhou et al. 2013; Jiang et al. 2013). SLs trigger proteasome-mediated degradation of D53 in a D14- and D3-dependent manner (Jiang et al. 2013; Zhou et al. 2013). KL908 with a mutation in the gene *D53* shares similar pleiotropism of phenotypes with other mutants *d3*, *d10*, *d14*, *d17*, and *d27*, including short stature, more tillers, and small panicles. These are considered as strigolactone-deficient or -insensitive mutants (Wei et al. 2006; Ishikawa et al. 2005; Gao et al. 2009).

Cytokinins are also regulators of dwarfism (Hirose et al. 2007; Wang et al. 2021b). One of the enzymes involved in cytokinin metabolism is OsCKX4, an oxidase/dehydrogenase involved in the degradation of cytokinins (Schmülling et al. 2003). Suppression of *OsCKX4* gene expression represses cytokinin catabolism, leading to reduced plant height. The discovery of cytokinins’ involvement and the associated molecular mechanisms in rice dwarfism was drawn from characterizing the cytokinin-deficient mutant *rice lateral branch* (*rlb*). The mutant carries a T-DNA insertion in the rice homeobox gene *OSH15*. Thus, *RLB* is an allele of *OSH15*. The protein RLB recruits PRC2 (Polycomb repressive complex 2) to epigenetically repress the transcription of *OsCKX4* by modifying H3K27me3, resulting in inhibition of *OsCKX4* transcription (Wang et al. 2021b). The *osh15(t)* from the *Shennong9816d* mutant is another naturally allelic variation of *OSH15*, showing a 30 bp insertion in intron 2 and a 1 bp mutation in exon 5 (Fan et al. 2016).

Initial evidence has also shown that the hormone abscisic acid may also play a role in rice dwarfism. The plant height of a near-isogenic rice line, NIL-Dd7, was shortened in comparison with its parent lines, but NIL-Dd7 did not respond to either GA or BR treatments. Recently, *Dd7* was mapped to a very short chromosomal fragment, and a candidate gene has been identified. The *Dd7* candidate gene contains an ORF encoding 9-cis-epoxycarotenoid dioxygenase 1, a key regulator of ABA synthesis (Dong et al. 2015; Zhao et al. 2018). However, more work is needed to confirm the role of ABA in regulating plant height (Zhao et al. 2018).

In addition to plant hormones, there are likely other mechanisms involved in regulating rice dwarf phenotypes. For example, increased abundance of the *OsmiR156h* microRNA in a natural allelic variant of the *sdt* gene reduced plant height but increased tillers and grain yield (Zhao et al. 2015). Other reported genes and proteins that affect plant height include α-tubulin (Sunohara et al. 2009) and a *methyladenine glycosylase* (a DNA repair enzyme) (Tong et al. 2016).

## Conclusion and Perspective

Rice is the staple food crop for more than half of the world's population and in more than 100 countries worldwide (Fukagawa and Ziska 2019). Rice breeding has experienced two quantum yield breakthroughs over the past three decades by introduction of the semi-dwarf gene *sd1* and hybrid technology (Siddiq and Vemireddy 2021; Peng et al. 2008). After these significant advances, rice yield has been in stagnation. In contrast, global demand for rice is predicted to increase by more than 70% of the current level. Global climate change, a growing population, and decreasing farmable lands pose serious challenges to satisfying rice demand in the future (Godfray et al. 2010; Siddiq and Vemireddy 2021, Food and Nations 2009). Cultural changes in rice farming such as rice direct seeding (DSR) and adoption of more advanced equipment and machines also require adaptable rice cultivars such as more lodging resistant varieties (Farooq et al. 2011; Hafeez-ur-Rehman et al. 2019). Thus, identification and use of new dwarfism and semi-dwarfism genes are needed to guarantee future food supply.

Although more than 60 dwarfing and semi-dwarfing genes have been identified through various technologies, most of these genes have serious defects in other agronomic traits along with reduced plant heights (Futsuhara and Kikuchi 1997; Gaur et al. 2020; Peng et al. 2021; Nagai et al. 2018; Wu et al. 2018). These unfavorable agronomic traits prevent their usefulness in practical rice breeding. More effort must be put into reducing these unfavorable traits associated with dwarfism so that the newly identified genes can be useful for increasing rice yield and quality.

In the past, our effort has focused on finding mutants from wild rice or generation of artificial mutants by chemical treatments or radiation. These efforts have been shown to be productive for unearthing useful genes for crop breeding, and we need to continue to enlarge our rice artificial mutant libraries in order to discover more dwarfing and semi-dwarfing genes. Since most dwarfing genes are in the pathways of plant hormone metabolism, disruption of the expression of these genes or mutations in key residues for normal three-dimensional structures of the encoded proteins will inevitably affect the normal functions of these genes. Future efforts shall also focus on generating mutants with small structural changes so that the main functions of these genes are not dramatically affected, but their functions for controlling plant heights are modified, resulting in shorter rice plants. Such elegant engineering is of course difficult, but possible with recent dramatic advances in new technologies, including precise genome sequencing, accurate tools for prediction of three-dimensional structures such as FoldAlpha2, targeted mutagenesis, and genome editing.

## Availability of Supporting data

All data and its supplementary information files of this study are included in this article.

## Supplementary Information

**Additional file 1: Table S1** Details on Rice dwarf and semi-dwarf genes from Rice Annotation Project Database (https://rapdb.dna.affrc.go.jp/).
